# Polymorphism of virulence genes and biofilm associated with in vitro induced resistance to clarithromycin in *Helicobacter pylori*

**DOI:** 10.1186/s13099-023-00579-4

**Published:** 2023-10-28

**Authors:** Naim Asyraf Rosli, Anis Rageh Al-Maleki, Mun Fai Loke, Eng Guan Chua, Mohammed Abdelfatah Alhoot, Jamuna Vadivelu

**Affiliations:** 1 Department of Medical Microbiology, Faculty of Medicine, Universiti Malaya, 50603 Kuala Lumpur, Malaysia; 2 Department of Medical Microbiology, Faculty of Medicine and Health Sciences, Sana’a University, Sana’a, Yemen; 3 Camtech Biomedical Pte Ltd, Singapore, Singapore; 4 School of Biomedical Sciences, Marshall Centre for Infectious Disease Research and Training, University of Western Australia, Perth, WA Australia; 5 Faculty of Pharmacy, Airlangga University, Surabaya, 60155 Indonesia; 6 School of Graduate Studies, Management & Science University, Shah Alam, Selangor Malaysia; 7 Medical Education Research and Development Unit, Faculty of Medicine, Universiti Malaya, Kuala Lumpur, Malaysia

**Keywords:** *Helicobacter pylori*, Whole genome sequencing, Virulence, Biofilm formation, Antibiotic resistance, Clarithromycin

## Abstract

**Background:**

Clarithromycin-containing triple therapy is commonly used to treat *Helicobacter pylori* infections. Clarithromycin resistance is the leading cause of *H. pylori* treatment failure. Understanding the specific mutations that occur in *H. pylori* strains that have evolved antibiotic resistance can help create a more effective and individualised antibiotic treatment plan. However, little is understood about the genetic reprogramming linked to clarithromycin exposure and the emergence of antibiotic resistance in *H. pylori*. Therefore, this study aims to identify compensatory mutations and biofilm formation associated with the development of clarithromycin resistance in *H. pylori*. Clarithromycin-sensitive *H. pylori* clinical isolates were induced to develop clarithromycin resistance through in vitro exposure to incrementally increasing concentration of the antibiotic. The genomes of the origin sensitive isolates (S), isogenic breakpoint (B), and resistant isolates (R) were sequenced. Single nucleotide variations (SNVs), and insertions or deletions (InDels) associated with the development of clarithromycin resistance were identified. Growth and biofilm production were also assessed.

**Results:**

The S isolates with A2143G mutation in the *23S rRNA* gene were successfully induced to be resistant. According to the data, antibiotic exposure may alter the expression of certain genes, including those that code for the Cag4/Cag protein, the vacuolating cytotoxin domain-containing protein, the sel1 repeat family protein, and the *rsmh* gene, which may increase the risk of developing and enhances virulence in *H. pylori*. Enhanced biofilm formation was detected among R isolates compared to B and S isolates. Furthermore, high polymorphism was also detected among the genes associated with biofilm production.

**Conclusions:**

Therefore, this study suggests that *H. pylori* may acquire virulence factors while also developing antibiotic resistance due to clarithromycin exposure.

**Supplementary Information:**

The online version contains supplementary material available at 10.1186/s13099-023-00579-4.

## Introduction

*Helicobacter pylori* is a spiral-shaped Gram-negative bacteria that thrives in the mucus and epithelial stomach mucosa of more than half of the world’s adult population [1]. It causes a variety of gastrointestinal disorders, such as gastritis, gastric ulcer, and duodenal ulcer, and is also associated with gastric cancer [2]. The standard triple therapy for *H. pylori* infection includes a proton pump inhibitor (PPI) and two antibiotics (amoxicillin with clarithromycin, or metronidazole) [3]. However, misuse and overuse of antimicrobials is causing the steady growth in antibiotic resistance and is severely hindering the eradication of *H. pylori*. Similarly, the success rates of clarithromycin as a component of the first-line therapy for *H. pylori* infections will continue to fall because of overuse of the antibiotic for the treatment of *H. pylori* [4, 5].

Clarithromycin is a bacteriostatic antibiotic that targets the peptidyl transferase loop of the V domain of the 23S ribosomal RNA (23S rRNA) molecule [6]. *H. pylori* clarithromycin resistance has been reported to be closely associated with point mutations in two neighbouring *23S rRNA* nucleotides, 2142 and 2143 [4], which reduces ribosome affinity for the macrolide, leading to enhanced resistance [7, 8]. However, other investigations revealed only 40–80% of clarithromycin resistant *H. pylori* had these *23S rRNA* point mutations [6, 9–12]. Consistently, the A2143G point mutation has been observed in both clarithromycin-sensitive and -resistant *H. pylori* [6, 13, 14]. Therefore, alternative mechanisms might play a role in clarithromycin resistance in *H. pylori *[10, 15, 16]. Biofilm formation, which is also a virulence mechanism, is also a potential resistance mechanism used by bacteria [17]. Bacteria protect themselves from host defence, disinfectants, and antibiotics by forming a biofilm. The bacteria in a biofilm are more resistant to antimicrobial agents and can exhibit a 10 to 1000-fold increase in antibiotic resistance compared to the same bacteria existing in a planktonic form [17, 18].

The prevalence of resistant patients with no prior history of clarithromycin-containing eradication treatment was 13.3% while resistance increased to 51.4% in previously treated patients as secondary (acquired) resistance [7]. Some *H. pylori* strains developed clarithromycin resistance in response to exposure to the antibiotic while others do not. We hypothesized that *H. pylori* strains that developed resistance to the antibiotic undergo specific genetic reprogramming and understanding these specific mutations can aid in determining a more effective and personalized antibiotic therapeutic regime. However, little is known about the genetic reprogramming associated with exposure to clarithromycin and antibiotic resistance development in *H. pylori*. Therefore, in this study, comparative genomic analysis was performed on clarithromycin-sensitive B isogenic isolates of *H. pylori* in comparison to their parental clarithromycin-sensitive clinical isolates and in vitro induced R isogenic isolates. Induced isolates collected one passage immediately prior to becoming clarithromycin-resistant were taken as the B isogenic isolates. Genetic alterations found in breakpoint isolates may not be directly associated to clarithromycin resistance, but they may serve to condition the organism to develop clarithromycin resistance. In addition, biofilm formed by these *H. pylori* isolates were compared to investigate for possible correlation between biofilm formation and exposure to clarithromycin or development of antibiotic resistance.

## Methodology

### Bacterial growth and culture conditions

*Helicobacter pylori* from the glycerol stocks of the clinical bacterial archival collection of the *Helicobacter* Research Laboratory (UM Marshall Centre) at the Universiti Malaya were used in this study. The *H. pylori* isolates were cultured on non-selective chocolate agar (CA) plates (Oxoid Ltd., UK) supplemented with 5% defibrinated horse blood and incubated at 37 °C in a 10% CO_2_ incubator for 3 days. To minimize the chance of mixed cultures, all the stock archival cultures (sweep cultures) were grown on selective CA plates supplemented with vancomycin (10 μg/mL) (Amresco Inc., Ohio), amphotericin B (5 μg/mL) (Bio-world Inc., USA), trimethoprim (5 μg/mL) (Santa Cruz Biotechnology Inc., USA), and nalidixic acid (20 μg/mL) (Bio-world Inc., USA) to obtain well-isolated colonies. Each of these well-isolated colonies are treated as individual clonal isolates and sub-cultured on fresh CA plates to get sufficient material. *H. pylori* was confirmed by rapid urease test, catalase test, oxidase test, and *16S rRNA* PCR using forward primer 5′-CTG GAG AGA CTA AGC CCT CC-3′ and reverse primer 5′-ATT ACT GAC GCT GAT TGT GC-3′ [19].

### Minimum inhibitory concentration (MIC)

The MICs of clarithromycin against *H. pylori* was determined on non-selective CA plate using MIC Test Strip (Calbiochem, Germany) according to the manufacturer’s instructions. Briefly, viable *H. pylori* colonies from non-selective CA plates grown for 3 days (72 h) were harvested and inoculated into the Brain Heart Infusion (BHI) broth. The turbidity of the suspension was adjusted by visual comparison to the McFarland standard no. 3, which is approximately ∼ 9.0 × 10^8^ CFU/mL and the suspension was spread onto a fresh non-selective CA plate with a clarithromycin-impregnated strip. The CA plates were incubated at 37 °C in a 10% CO_2_ incubator for 3 days. A drop-shaped inhibition zone intersects the graded test strip at the MIC of the antibiotic. The experiments were performed in triplicate with 3 biological replicates. Based on European Committee on Antimicrobial Susceptibility Testing (EUCAST) standards (version 13.0), the MIC breakpoint for clarithromycin is > 0.25 μg/mL. Clarithromycin sensitive isolates from the collection were selected for induction experiment.

### In vitro clarithromycin resistance induction

Clarithromycin-sensitive *H. pylori* isolates were induced by the method according to Yan et al*.* [20] with modifications. Briefly, *H. pylori* isolates were exposed to incrementally doubling concentrations of clarithromycin from 0.0156 to 32 µg/mL incorporated into CA plate. To ensure that the induced strains were stable, MICs of the R, B and S isolates were confirmed using MIC Strip Test as previously described after ten passages of the R isolates on non-selective CA plate to determine the stability of resistance and after storage frozen at − 80 °C for 3–5 months (Additional file 2 and Additional file 3: Fig. S1).

### Random amplification of polymorphic DNA-polymerase chain reaction (RAPD-PCR)

The identity between resistant strains and their corresponding parental sensitive strains before induction were verified by RAPD-PCR typing using primers as previously described [21]. The primers were 1254 5′-CCG CAG CCA A-3′, 1281 5′-AAC GCG CAA C-3′ and 1283 5′-GCG ATC CCC A-3′. The conditions for PCR amplification were denaturation at 95 °C for 3 min, followed by 45 cycles of denaturation at 95 °C for 1 min, annealing at 36 °C for 1 min, and extension at 72 °C for 1 min: and then a final extension at 72 °C for 5 min.

### Sanger sequencing

The bacteria were grown for 3 days in 10 mL of BHI broth supplemented with 1% ß-cyclodextrin and 0.4% yeast extract and incubated at 37 °C in a 10% CO_2_ incubator. To collect the pellet, the bacterial broth was centrifuged for 10 min at 8000 rpm. The DNA of S, B and R isolates were extracted using the MasterPure™ Complete DNA and RNA Purification Kit (Lucigen, USA) and used for Sanger sequencing as well as whole genome sequencing. Two primers corresponding to bases 1820–1839 [Hp23-1: 5′-CCACAGCGATGTGGTCTCAG-3′] and from positions 2244–2225 [Hp23-2: 5′-CTCCatAAGAGCCAAAGCCC-3′] flanking a region of 425 bp within bacterial *23S rRNA* peptidyl transferase as described by Ho et al. [22]. The PCR amplified products were sequenced on a ABI PRISM 3730xl Genetic Analyzer (Applied Biosystems, USA) by 1st Base (Singapore). Multiple sequence alignment was done by Bioedit version 7.2.5 and CodonCode Aligner version 10.0*.* The sequences were compared with the *23S rRNA* of the reference genome (*H. pylori* UM 032 and *H. pylori* 26695).

### *Library preparation*, *and sequencing*

The extracted DNA was used in library preparation. Following the manufacturer’s instructions, preparations for next-generation sequencing libraries were constructed using VAHTS Universal Pro DNA Library Prep Kit for Illumina V1. For each sample, 200 μg genomic DNA was randomly fragmented by Covaris ultrasonication system to an average size of 300–350 bp. The fragments underwent treatment with End Prep Enzyme Mix for end repairing, 5′ Phosphorylation and 3′ adenylated, to add adaptors to both ends. Next, DNA Cleanup beads selected the size of the adaptor-ligated DNA. Then, using P5 and P7 primers, each sample was then amplified by PCR for 8 cycles, with both primers carrying sequences which can anneal with flowcell to perform bridge PCR and P7 primer carrying a six-base index allowing for multiplexing. An Agilent 2100 Bioanalyzer was used to clean up and validate the PCR products. The qualified libraries were sequenced pair end PE150 (V1) on the Illumina Novaseq 6000.

### Single-nucleotide variations (SNVs) and insertion and deletions (InDels) identification

The sequences of adaptors, PCR primers, content of N bases greater than 10%, and bases of poorer quality than 20 were removed using Cutadapt (V1.9.1). Using BWA (V0.7.17), clean data were mapped to the reference genome (*H. pylori* UM032, CP005490.3). Mapping results were processed by Picard (V2.25.7) to remove duplication. The HaplotypeCaller calls SNV/InDel with GATK (V3.8.1) software. Annotation for SNV/InDel was performed by Annovar (V21 Apr 2018).

### Probability of mutation occurrence

The mutation rate of a gene was calculated by dividing the number of mutations (identified SNVs and InDels) within the gene with the number of base pair of the gene. Meanwhile, each strain’s threshold was calculated by dividing the total number of mutations within the strain with the total number of base pairs of the genome. The probability of occurrence of these mutations was computed by dividing the rate of mutation of the gene with the threshold of the corresponding strain.

### Growth curve

The growth pattern of the *H. pylori* strains was performed according to Al-Maleki et al*.* [23]. Briefly, 12 isolates of *H. pylori* of S, B and R were cultured in BHI broth (Oxoid, UK) supplemented with 1% ß-cyclodextrin and 0.4% yeast extract in a 24-well plate and incubated at 37 °C in a 10% CO_2_ incubator. The optical density (OD_600 nm_) of the bacterial broth suspension was standardized to optical density (OD_600 nm_) of 0.02 using spectrophotometer (Thermo Fisher Scientific, USA) at t = 0 h. Bacterial broth suspension was collected and the OD_600 nm_ is measured every 24 h over 7 days. Then, a serial tenfold dilutions in 1× sterile phosphate buffer saline (PBS) was performed and subsequently plated on CA plate followed by incubation at 37 °C in a 10% CO_2_ incubator for 3 days. The viable count was then performed to calculate the CFU which represent the number of living cells in the broth at every time point. Growth curve was performed as three independent replicates.

### Biofilm formation

The inhibition of biofilm formation was assessed using methods that were described previously [24]. Briefly, *H. pylori* cultured on CA plate for 3 days in a 10% CO_2_ incubator were harvested and inoculated in BHI broth supplemented with 1% β-cyclodextrin and 0.4% yeast extract in a 24-well plate (Corning, USA) for another 3 days. The bacterial suspension was adjusted to 1–2 × 10^8^ CFU/mL. The development of the biofilm was visually inspected at days 3, 5, and 7. The amount of biofilm produced was measured after day 7 using 0.1% crystal violet staining. After 30 min of gentle agitation at 100 rpm, the unbound crystal violet was removed. The biofilm was destained with a 19:1 ethanol-acetic acid solution after the crystal violet-treated wells were washed with distilled water. The solution collected was measured at OD_595 nm_ on a spectrophotometer. The amount of crystal violet absorbed by the biofilm was determined by taking the mean absorbance value. The experiment was performed in triplicate.

### Statistical analysis

IBM SPSS statistics version 22 software was used to perform the statistical analyses for growth curve and biofilm formation assays. One-way Analysis of Variance (ANOVA) and two-sample t-test were used to compare the means between variants. A *p*-value < 0.05 was considered statistically significant.

## Results

### Clarithromycin-resistant isogenic isolates

In total, 86 *H. pylori* clinical isolates collected from patients presenting for endoscopy at the Universiti Malaya Medical Centre between November 2011 and January 2015 were screened for clarithromycin resistance. Based on EUCAST resistant breakpoint of > 0.25 μg/mL, 60 isolates (69.77%) were susceptible to clarithromycin and 26 isolates (30.23%) were resistant to the antibiotic. Twenty randomly selected clarithromycin-sensitive *H. pylori* clinical isolates and one standard strain (NCTC 11637) were inducted by exposure to incrementally doubling concentrations of clarithromycin (Table 1 and Additional file 2). After 10 to 12 passages on clarithromycin CA plates, four clarithromycin-sensitive *H. pylori* isolates were successfully induced in vitro to become resistant with a success rate of 19.0% (Table 2). Clarithromycin CA plates were used to continue the induction until the isolates showed resistance to > 64 µg/mL on the MIC Test Strip. Notably, all the four successfully induced isolates originally harbour the A2143G variant despite been phenotypically susceptible to clarithromycin. On the other hand, the remaining 17 clarithromycin-sensitive isolates did not harbour the A2143G variant and were not successfully induced by exposure to the antibiotic.

**Table 1:** Twenty randomly selected clarithromycin-sensitive *H. pylori* clinical isolates and one standard strain were inducted by exposure to clarithromycin

No	Isolate	CLR sensitivity/resistance	CLR MIC (µg/mL)	*23S rRNA* (A2142G/C)	*23S rRNA* (A2143G)	Induction success
1	NCTC 11637	S	0.032	No	No	No
2	UM025B	S	0.032	No	No	No
3	UM067B SW	S	0.125	No	No	No
4	UM113B	S	0.125	No	No	No
5	UM113B C5	S	0.032	No	No	No
6	UM114	S	0.032	No	No	No
7	UM171	S	0.064	No	Yes	Yes
8	UM276 R (71)	S	0.032	No	No	No
9	UM276 S (53)	S	0.032	No	No	No
10	UM303A C6	S	0.032	No	No	No
11	UM408 SW	S	0.032	No	No	No
12	UM411 SW	S	0.032	No	No	No
13	UM443A (33)	S	0.032	No	No	No
14	UM443A SW	S	0.032	No	No	No
15	UM622A1^a^	S	0.032	No	No	No
16	UM626A1	S	0.064	No	Yes	Yes
17	UM650B	S	< 0.016	No	Yes	Yes
18	UM663B2	S	0.064	No	No	No
19	UM678A	S	0.064	No	Yes	Yes
20	UM692B2	S	0.032	No	No	No
21	UM113A SW	S	0.032	No	No	No
22	*H. pylori* 26695	S	0.016	No	No	Not Done
23	UM032	S	0.016	No	No	Not Done
24	UM202	R	–	No	No	Not Done
25	UM233	S	–	No	No	Not Done
26	UM370	R	–	No	Yes	Not Done

^a^The isolate number consist of: UM stands for Unversiti Malaya, followed by isolate number, letter designations (A: Antrum, B: Body, C: Cardia, SW: Sweep), and the number of clonal isolates

**Table 2:** MICs of *H. pylori* isolates before, and after in vitro induction with clarithromycin

No	Isolate	MIC in µg/mL (passage number)			
		S isolates before induction	B isolates at breakpoint	R isolates after breakpoint^a^	R isolates after resistance induction^b^
1	UM171	0.064 (P0)	0.125 (P4)	1 (P5)	> 64 (P10)
2	UM626A1	0.064 (P0)	0.125 (P5)	2 (P6)	> 64 (P11)
3	UM650B	< 0.016 (P0)	0.125 (P7)	1 (P8)	> 64 (P12)
4	UM678A	0.064 (P0)	0.125 (P5)	1 (P6)	> 64 (P10)

^a^Passage number when isolate developed resistance for the first time

^b^Passage number when there is no inhibition zone using MIC Test Strip

### Stability of resistance

**Fig. 1:**
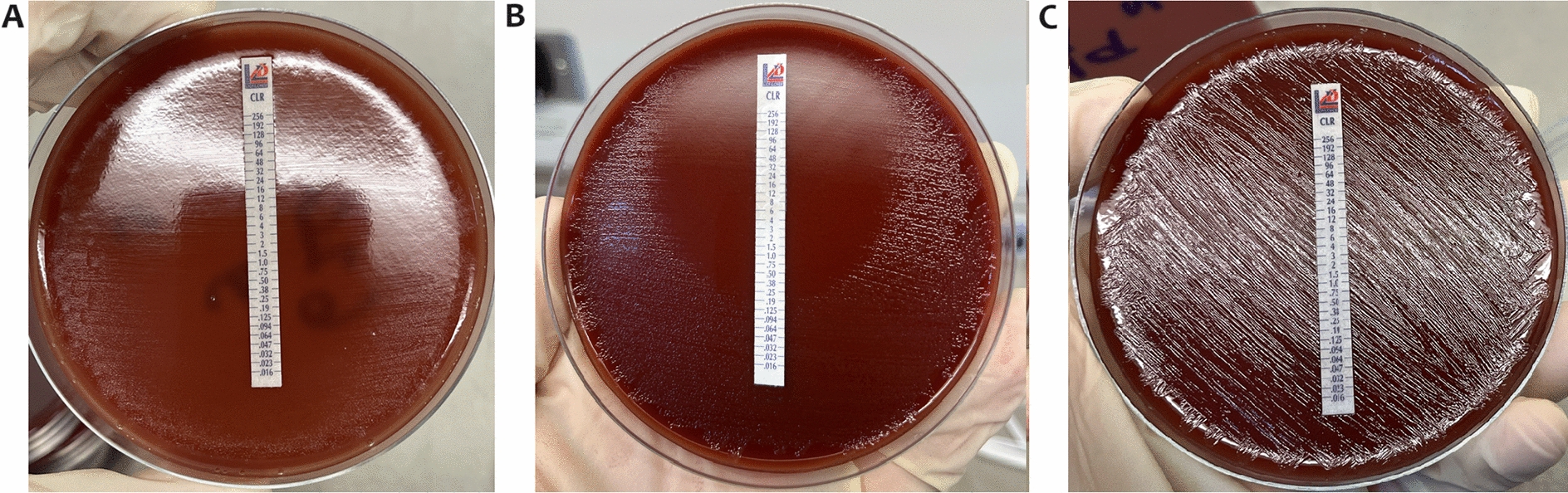
MIC Test Strip results for induced resistant *H. pylori* R isogenic isolates on CA plate to determine the stability of the resistance. **A** S parental isolates, **B** B isolates collected one passage immediately prior to becoming clarithromycin resistant, and **C** R isolates after 10 successful rounds of growth on non-selective CA plate

All the four R isogenic isolates maintained their MICs of > 64 µg/mL against clarithromycin after 10 successful rounds of growth on non-selective CA plate, and after storage frozen at − 80 °C for 3–5 months in a BHI broth with 20% glycerol. The MICs were confirmed using MIC Test Strip (Fig. 1). These induced isolates probably underwent stable genetic reprogramming that contributed to the persistence of antibiotic resistance even in the absence of selective pressure.

### RAPD genotypes

**Fig. 2:**
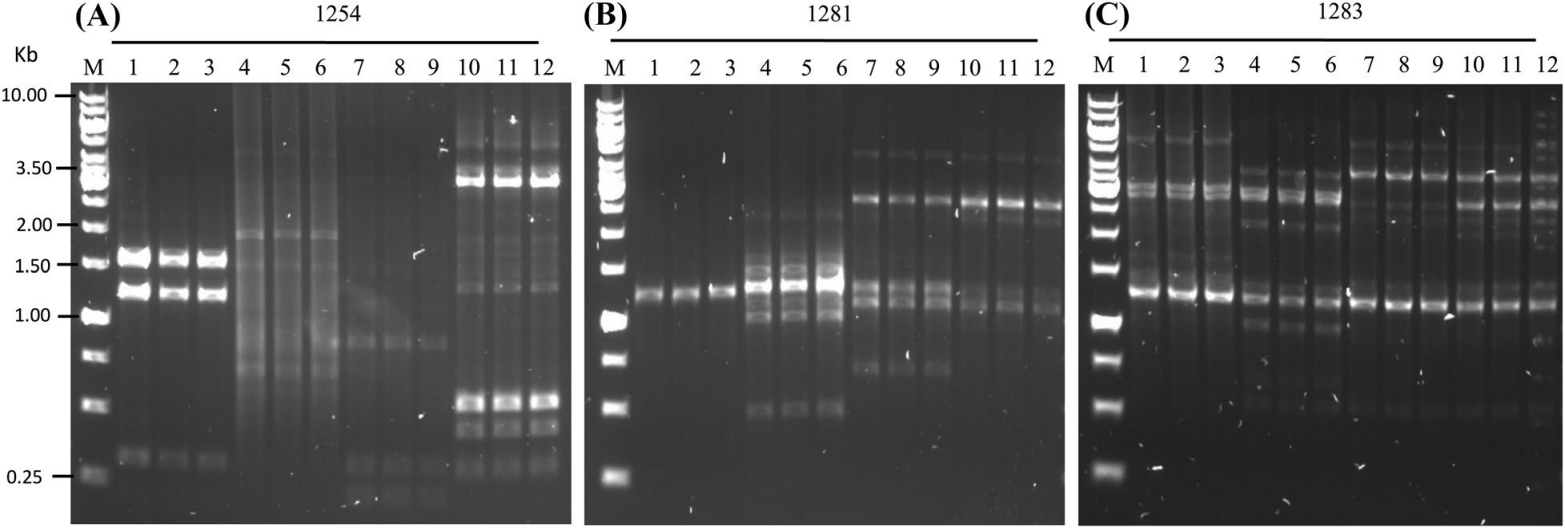
Agarose gel electrophoresis of a RAPD-PCR typing of *H. pylori* isolates*.* S, B, and R Bands were electrophoresed using 1.0% agarose gel (1 h, 5 V/cm, 1XTris Acetate-EDTA buffer) and visualized by cybersafe staining. Marker (M): 1 kb ladder marker (Fermentas, USA). **A** (RAPD 1, 1254 primers), **B** (RAPD 2, 1281 primers), and **C** (RAPD 3, 1283 primers). Lanes 1–3: UM171 (S, B, and R), Lanes 4–6: UM626A1 (S, B, and R), Lanes 7–9: UM650B (S, B, and R), Lanes 10–12, UM678A (S, B, and R)

RAPD PCR was performed to verify the identity of S, B, R isolates. Based on Fig. 2, the RAPD analysis of the four parental S isolates were not related, each isolate showed a distinctive pattern of bands. In addition, the B and R isogenic isolates were identical in genotype to their respective parental S isolates.

### *23SrRNA* genotypes and resistance to clarithromycin

**Fig. 3:**
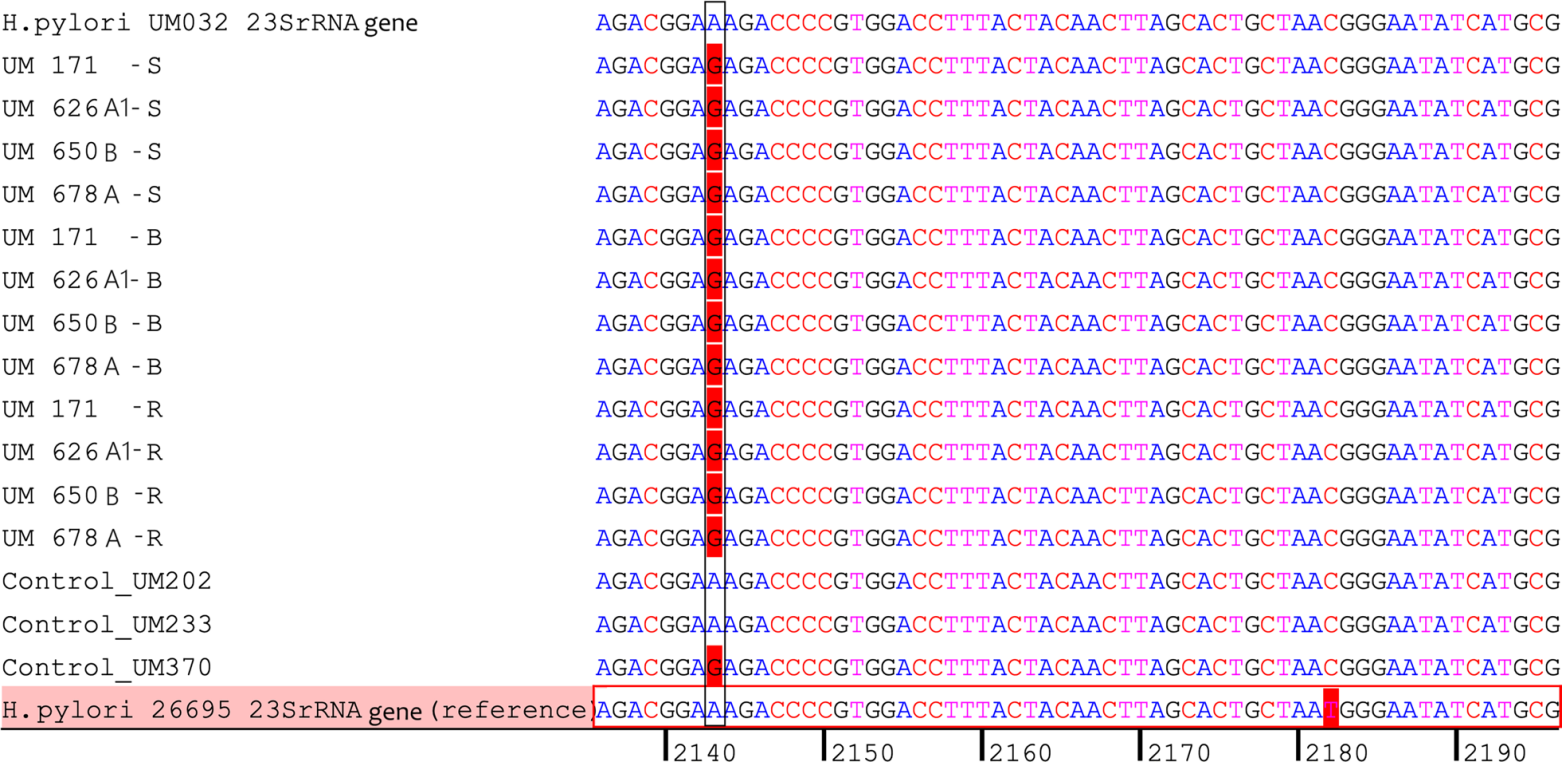
Sequence alignment of the *23S rRNA* gene of *H. pylori*. The base sequence of the *23S rRNA* gene fragment of the twelve isolates (UM171, UM626A1, UM650B, and UM678A; S, B, and R) aligned with the base sequence of the reference *H. pylori* UM032. Position 2143 is highlighted. Multiple sequence alignment was performed using Bioedit version 7.2.5 and CodonCode Aligner version 10.0*.*3. *H. pylori* 26695, UM032, and UM233 were susceptible to clarithromycin while *H. pylori* UM202 and UM370 were resistance to clarithromycin. Common point mutations in A2142G/C and A2143G positions in the *23S rRNA* gene of *H. pylori* 26695 (ATCC 700392), reference strain (UM032), two naturally clarithromycin resistant strains (UM202 and UM370), and a non-induced sensitive strain (UM233) were also included to show the pattern of mutations in comparison to that of the induced isolates

The S, B, and R isolates were tested for clarithromycin susceptibility, and the results showed that all S and B isolates were susceptible while all the induced R isolates were resistant to clarithromycin. Interestingly, despite been susceptible to clarithromycin, all four S parental isolates had A2143G variation of *23S rRNA* (Fig. 3). There were no variations in positions 2142 and 2143 of the gene between the parental isolates and the induced isogenic isolates. The *23S rRNA* Sanger sequencing datasets supporting the conclusions of this article are available in the NCBI’s GenBank repository under the accession OR357686-OR357715.

### Quality of *H. pylori* genomes

After trimming the low-quality reads, there were 19 to 36 million cleaned reads. Cleaned reads from S, B, and R samples were directly mapped to the UM032 reference *H. pylori* genome. *H. pylori* UM032 was chosen as the reference genome for mapping because UM032 genome was fully sequenced, extensively studied, and was derived from *H. pylori* isolated in the same human population. Sequencing coverage and average depth ranged from 95.08 to 95.47% and from 1152.47 to 2151.57, respectively. Therefore, the efficient reads were sufficient for SNV/InDel analysis. SNV/InDel analysis of the B and R isolates was carried out with reference to the corresponding S isolates. The number of identified SNVs ranged from 5300 to 35,742 and InDels ranged from 646 to 2679. The WGS sequencing datasets supporting the conclusions of this article are available in the NCBI’s Sequence Read Archive (SRA) database repository under the accession ID PRJNA999133, unique persistent identifier and hyperlink to datasets in https://www.ncbi.nlm.nih.gov/sra/PRJNA999133.

### Clarithromycin resistance-associated variants

#### SNV and InDel

The SNV and InDel analysis of the corresponding S isolates was used to compare the SNV and InDel of the B and R isolates. A total of 67,688 SNV mutations were detected among the B isolates and 65,476 SNV mutations were detected among the R isolates. Moreover, 5442 InDel mutations detected among the B isolates, and 5244 InDel mutations detected among the R isolates (Table 3).

**Table 3:** Classification of SNVs and InDels

Isolate	Group	Isolate ID	SNV				InDel			
			Non-synonymous^a^	Synonymous^a^	Stop gain	Stop loss	Non-frameshift deletion	Frameshift deletion	Non-frameshift insertion	Frameshift insertion
UM171	B	UM171-B	7006	15,900	71	18	133	420	115	409
	R	UM171-R	6764	15,358	70	19	136	411	112	395
UM626A1	B	UM626A1-B	9101	21,425	83	27	169	510	135	485
	R	UM626A1-R	8635	20,422	78	27	148	465	139	443
UM650B	B	UM650B-B	1432	2978	20	3	52	136	38	139
	R	UM650B-R	1504	3108	18	3	53	136	40	143

The corresponding S isolates were used as reference

^a^Those numbers were detected after mapping against reference strains (UM32) for the purpose of annotation

Among of all identified variations in B and R, 119,432 were in the coding region, 1888 were located in the intergenic region, 4995 were located in the upstream, 7827 were located in the downstream, 9702 were located in the upstream; downstream, and 6 were located in the UTR5 (Table 4).

**Table 4:** Distribution of SNV/InDel on genome

Isolate	Group	Isolate ID	Categories					
			Exonic	Intergenic	Upstream	Downstream	Upstream; downstream	UTR5^a^
UM171	B	UM171-B	24,072	320	953	1536	1881	1
	R	UM171-R	23,265	327	938	1479	1898	0
UM626A1	B	UM626A1-B	31,935	559	1352	2032	2541	2
	R	UM626A1-R	30,357	533	1375	1998	2454	2
UM650B	B	UM650B-B	4798	61	202	440	445	0
	R	UM650B-R	5005	88	175	342	483	1

The corresponding S isolates were used as reference

^a^UTR5: untranslated reg

### Genes associated with virulence and antibiotic resistance

Genes with the highest rate of mutation in response to clarithromycin may have a higher likelihood of been associated directly (causation) or indirectly (compensation) with clarithromycin resistance. An additional file shows this in more detail of the genes with high number of mutations (see Additional file 1). Interestingly, mutations above threshold of > 1 were detected in genes that play a role in virulence and survival (*cag4*, *rsmH*, gene encoding sel1 repeat family protein, and gene encoding vacuolating cytotoxin domain-containing protein) in the induced isolates (B and R) against their corresponding S isolates (Table 5). Additionally, the specific mutations of these genes were also noted which may associate with the development of antibiotic resistance in *H. pylori* in response to clarithromycin (Table 6).

**Table 5:** Rate of mutations of genes associated with clarithromycin resistance development in the UM171, UM626A1, and UM650B

Locus tag	Protein ID	Protein name	Isolate	Rate of mutation	Threshold	Probability of mutation occurrence
K747_RS06340	WP_015644956.1	Sel1 repeat family protein	UM171-B	0.1054	0.0180	5.8387
			UM171-R	0.0948	0.0175	5.4160
			UM626A1-B	0.0948	0.0241	3.9339
			UM626A1-R	0.0948	0.0230	4.1162
			UM650B-B	0.0141	0.0037	3.7658
			UM650B-R	0.0070	0.0038	1.8372
K747_RS06360	WP_015644959.1	Transglycosylase SLT domain-containing protein (Cag4)	UM171-B	0.0180	0.0180	6.4219
			UM171-R	0.0175	0.0175	5.3848
			UM626A1-B	0.0241	0.0241	3.8298
			UM626A1-R	0.0230	0.0230	4.0073
			UM650B-B	0.0037	0.0037	16.3223
			UM650B-R	0.0038	0.0038	15.9259
K747_RS06805	WP_015645027.1	Vacuolating cytotoxin domain-containing protein	UM171-B	0.0568	0.0180	3.1475
			UM171-R	0.0509	0.0175	2.9048
			UM626A1-B	0.0586	0.0241	2.4298
			UM626A1-R	0.0591	0.0230	2.5651
			UM650B-B	0.0182	0.0037	4.8890
			UM650B-R	0.0215	0.0038	5.6153
K747_RS07265	WP_015645103.1	16S rRNA (cytosine(1402)-*N*(4))-methyltransferase (RsmH)	UM171-B	0.0335	0.0180	1.8547
			UM171-R	0.0432	0.0175	2.4666
			UM626A1-B	0.0443	0.0241	1.8364
			UM626A1-R	0.0292	0.0230	1.2654
			UM650B-B	0.0076	0.0037	2.0259
			UM650B-R	0.0108	0.0038	2.8239

**Table 6:** Specific mutations associated with clarithromycin resistance development in UM171, UM626A1, and UM650B

Type	Locus tag (gene)/protein	Position	Type of variation	Bases change (amino acid change)								
				UM171			UM626A1			UM650B		
				S	B	R	S	B	R	S	B	R
SNV	K747_RS06360 (*cag4*)/transglycosylase SLT domain-containing protein	NC_021215.3:1292240	Non-synonymous, exonic	G (R)	G>A (R47K)	G>A (R47K)	G (R)	G>A (R47K)	G>A (R47K)	G (R)	G>A (R47K)	G>A (R47K)
		NC_021215.3:1292491	Non-synonymous, exonic	G (V)	G>A (V131M)	G>A (V131M)	G (V)	G>A (V131M)	G>A (V131M)	G (V)	G>A (V131M)	G>A (V131M)
		NC_021215.3:1292500	Non-synonymous, exonic	A (I)	A>T (I134L)	A>T (I134L)	A (I)	A>T (I134L)	A>T (I134L)	A (I)	A>T (I134L)	A>T (I134L)
	K747_RS07265*(rsmH)*/16S rRNA (cytosine(1402)-*N*(4))-methyltransferase	NC_021215.3:1499504	Non-synonymous, exonic	T (S)	T (S)	T>G (S244A)	T (S)	T>G (S244A)	T>G (S244A)	T (S)	T (S)	T>G (S244A)
		NC_021215.3:1499056	Non-synonymous, exonic	T (N)	T (N)	T>A (N94K)	T (N)	T>A (N94K)	T>A (N94K)	T (N)	T>A (N94K)	T>A (N94K)
		NC_021215.3:1499175	Non-synonymous, exonic	A (D)	A (D)	A>G (D134G)	A (D)	A>G (D134G)	A>G (D134G)	A (D)	A>G (D134G)	A>G (D134G)
		NC_021215.3:1499345	Non-synonymous, exonic	C (L)	C (L)	C>T (L191F)	C (L)	C>T (L191F)	C>T (L191F)	C (L)	C>T (L191F)	C>T (L191F)
	K747_RS06340/Sel1 repeat family protein	NC_021215.3:1289818	Non-synonymous, exonic	C (D)	C>T (D39N)	C>T (D39N)	C (D)	C>T (D39N)	C>T (D39N)	C (D)	C>T (D39N)	C>T (D39N)
InDel	K747_RS06360 (*cag4*)/transglycosylase SLT domain-containing protein	NC_021215.3:1292229_1292232	Frameshift deletion, exonic	AGTG (E)	– (E43fs)	AGTG (E)	AGTG (E)	– (E43fs)	– (E43fs)	AGTG (E)	– (E43fs)	– (E43fs)
		NC_021215.3:1292233_1292234	Frameshift insertion, exonic	– (V)	ATAA (V45fs)	– (V)	– (V)	ATAA (V45fs)	ATAA (V45fs)	– (V)	ATAA (V45fs)	ATAA (V45fs)
	K747_RS06805/vacuolating cytotoxin domain-containing protein	NC_021215.3:1393215_1393219	Frameshift deletion, exonic	ACTAG (L)	– (L2430fs)	ACTAG (L)	ACTAG (L)	– (L2430fs)	– (L2430fs)	ACTAG (L)	– (L2430fs)	– (L2430fs)
		NC_021215.3:1393222_1393223	Frameshift insertion, exonic	– (S)	CAAAC (S2433fs)	– (S)	– (S)	CAAAC (S2433fs)	CAAAC (S2433fs)	– (S)	CAAAC (S2433fs)	CAAAC (S2433fs)

### Growth curves

**Fig. 4:**
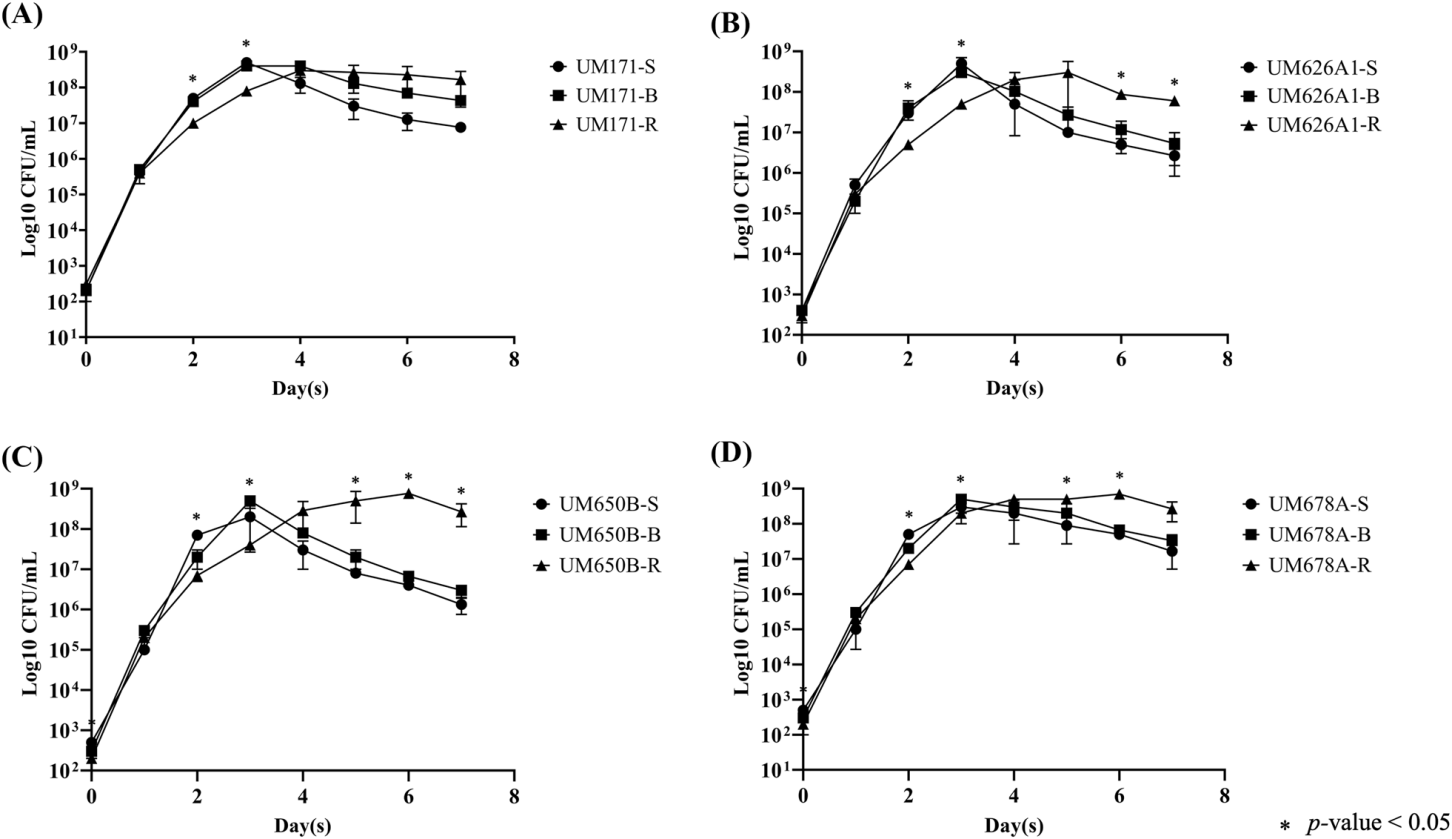
Growth curves of *H. pylori* S, B, and R isolates. The cultures were inoculated in BHI broth supplemented with 1% β-cyclodextrin and 0.4% yeast extract and incubated at 37 °C in a 10% CO_2_ incubator. The viable count was performed to calculate the CFU counts which represent the number of living cells in the broth at every 24 h over 7 days. A One-Way ANOVA in SPSS (version 22) was used to compare the means between variants

The growth rate of the *H. pylori* S, B, and R isolates were comparable within the initial 1 day of growth. However, S and B isolates showed an increase in growth compared to R isolate from 2 to 3 days of growth (Fig. 4). The OD_600 nm_ of S, B, and R were found to be associated well with the bacterial viable count.

### Biofilm assessment

**Fig. 5:**
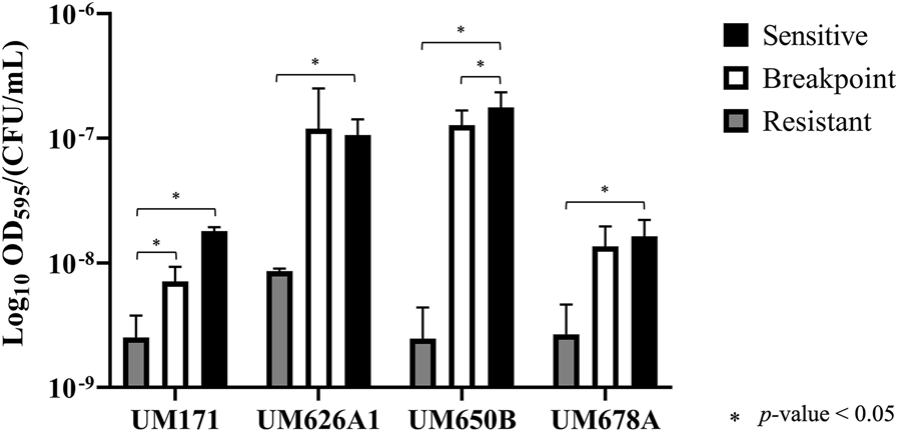
The average biofilm development of *H. pylori* isolates was divided against growth level of day 7. S, B, and R strains were inoculated in BHI broth supplemented with 1% β-cyclodextrin and 0.4% yeast extract. The amount of biofilm produced was measured after day 7 using 0.1% crystal violet staining. Two-sample t-test was used to calculate the *p*-values and *p-*value < 0.05 was taken as statistically significant as indicated by “*”

The multicellular survival tactic of biofilm formation, which occurs at the population level, indirectly improves the fitness of bacteria for overall survival. In this study, the R isolates produced significantly (*p*-value < 0.05, > twofold changes) more biofilm compared to S. Meanwhile, B isolates produced more biofilm compared to S isolates (Table 7). The average biofilm development of *H. pylori* isolates on day 7 was then divided against their corresponding growth level of day 7. The results showed that the R isolates produced significantly (*p*-value < 0.05) more biofilm compared to S. Meanwhile, B isolates produced more biofilm compared to S isolates but was only statistically significant (*p*-value < 0.05) for UM171 (Fig. 5).

**Table 7:** Average *H. pylori* biofilm formed on day 7

Isolate	Group	Sample ID	Average OD_595 nm_ reading	Fold difference		
				B vs S (*p*-value)	R vs S (*p*-value)	R vs B (*p*-value)
UM171	S	UM171-S	0.14	2.07 (< 0.05)	2.36 (< 0.05)	1.14 (≥ 0.05)
	B	UM171-B	0.29			
	R	UM171-R	0.33			
UM626A1	S	UM626A1-S	0.22	1.39 (< 0.05)	2.03 (< 0.05)	1.45 (< 0.05)
	B	UM626A1-B	0.36			
	R	UM626A1-R	0.49			
UM650B	S	UM650B-S	0.21	1.86 (< 0.05)	2.19 (< 0.05)	1.18 (< 0.05)
	B	UM650B-B	0.40			
	R	UM650B-R	0.47			
UM678A	S	UM678A-S	0.19	1.95 (< 0.05)	2.63 (< 0.05)	1.35 (< 0.05)
	B	UM678A-B	0.38			
	R	UM678A-R	0.52

### Identification of genes associated with biofilm formation

The presence of annotated genes in the *H. pylori* genomic sequences of B and R mutants was shown to be substantially linked with the capacity to build biofilm (Table 8). It is interesting to note that bacteria in biofilms frequently display mutator phenotypes and phenotypic variety, indicating that genetic instability and mutation are key components of biofilm formation. Interestingly, mutations were found in the B and R mutants. These included the genes *hypE*, *hypF*, and gene encoding cag pathogenicity island (Table 8).

**Table 8:** Mutations associated with biofilm formation in UM171, UM626A1, and UM650B

Type	Locus tag (gene)/protein	Position	Type of variation	Bases change (amino acid change)								
				UM171			UM626A1			UM650B		
				S	B	R	S	B	R	S	B	R
SNV	K747_RS04025*(hypE)/*hydrogenase expression/formation protein HypE	NC_021215.3:820809	Non-synonymous, exonic	C (G)	C>G (G145A)	C>G (G145A)	C (G)	C>G (G145A)	C>G (G145A)	C (G)	C>G (G145A)	C>G (G145A)
		NC_021215.3:821112	Non-synonymous, exonic	T (H)	T>C (H44R)	T>C (H44R)	T (H)	T>C (H44R)	T>C (H44R)	T (H)	T>C (H44R)	T>C (H44R)
		NC_021215.3:821113	Non-synonymous, exonic	G (H)	G>T (H44N)	G>T (H44N)	G (H)	G>T (H44N)	G>T (H44N)	G (H)	G>T (H44N)	G>T (H44N)
	K747_RS04030(*hypF*)/carbamoyltransferase HypF	NC_021215.3:822687	Non-synonymous, exonic	C (A)	C>T (A271T)	C>T (A271T)	C (A)	C T (A271T)	C>T (A271T)	C (A)	C>T (A271T)	C>T (A271T)
		NC_021215.3:822837	Non-synonymous, exonic	C (D)	C>T (D221N)	C>T (D221N)	C (D)	C>T (D221N)	C>T (D221N)	C (D)	C>T (D221N)	C>T (D221N)
	K747_RS06380/Cag pathogenicity island protein	NC_021215.3:1300640	Non-synonymous, exonic	T (K)	T>G (K608Q)	T>G (K608Q)	T (K)	T>G (K608Q)	T>G (K608Q)	T (K)	T>G (K608Q)	T>G (K608Q)
		NC_021215.3:1300687	Non-synonymous, exonic	C (S)	C>T (S592N)	C>T (S592N)	C (S)	C>T (S592N)	C>T (S592N)	C (S)	C>T (S592N)	C>T (S592N)

## Discussions

The global resistance rate of clarithromycin increased significantly from 24.28% in 2010–2017 to 32.14% in 2018–2021 with Switzerland, Portugal, and Israel having the highest resistance rate [25]. Similarly, clarithromycin resistance in Malaysia is also increasing from 6.8% between July 2011 and August 2012 [26] to 35.6% between April 2014 and August 2015 [27]. Thus, constantly monitoring clarithromycin-resistant rates of *H. pylori* is crucial for making informed decision of the most appropriate eradication therapies with good clinical outcomes. In this study, the rate of clarithromycin resistance was estimated to be 29.9%. However, this must be interpreted with caution as different resistance breakpoint and testing methods were used by different researchers in this field. Notably, Hanafiah et al*.* [27] and our data did not distinguish primary and secondary resistance cases while the earlier study [26] had excluded all known cases of treatment failure.

*Helicobacter pylori* clarithromycin resistance is mostly caused by point mutations (A2142G/C and A2143G) in the *23S rRNA* gene’s peptidyl transferase loop region [28]. This mutation in *23S rRNA* gene (A2143G) has also been observed in clarithromycin-sensitive and clarithromycin-resistant *H. pylori* strains by other researchers. Zhang et al*.* [13] has detected the A2143G mutation in 45.5% (5/11) clarithromycin-sensitive strains. Moreover, A2143G mutation was also found in two out of six clarithromycin-sensitive *H. pylori* strains in China [6]. Similarly, sensitive strains with A2143G mutation were also reported previously among *H. pylori* stains in Mexico [14]. In this study, sequencing of clarithromycin-sensitive *H. pylori* detected mutation A2143G mutations of the *23S rRNA* in four out of 26 isolates. Interestingly, these four isolates, which were phenotypically clarithromycin-sensitive but harbour the A2143G, were successfully induced to clarithromycin resistant by exposure to the antibiotic in vitro. On the other hand, none of the other clarithromycin-sensitive isolates could be induced. Additionally, sensitive strains could carry silent antimicrobial resistance genes, often known as cryptic genes. Bacteria may carry these silent genes on their chromosomal DNA or plasmids but do not show the appropriate phenotypic antibiotic resistance [29, 30]. The majority of strains with silent genes are clinical strains [31]. Several Gram-negative bacteria have been reported to carry cryptic genes [32–37]. In each of these cases, the genes’ promoter and resistance gene sequences were intact, indicating that the process of silencing is not well understood. This implies that under some circumstances, it is possible for genes to spread silently throughout bacterial populations. Such silent genes “off status” may express phenotypic resistance “on status” when they are subjected to selective pressure, such as pressure from antibiotics. It was previously observed by Stasiak et al*.* [36] that antibiotic pressure can cause the activation of silent antimicrobial genes. Moreover, *H. pylori* possesses regulatory genes that regulate the expression of various antibiotic resistance genes [38]. Therefore, investigating the gene expression linked to antibiotic resistance can reveal insights into the mechanisms *H. pylori* employ to survive antibiotic treatment. Therefore, we hypothesized that these clarithromycin sensitive strains with A2143G mutation in the present study were potentially resistant to clarithromycin and resistance were “switched on” when exposed to the antibiotic.

On the other hand, the result of WGS showed changes in genes associated with virulence and antibiotic resistance and may influence in the development of clarithromycin resistance among *H. pylori* strains. Although four pairs of induced resistant isolates were sequenced, based on the quality control (QC) data, UM678A was excluded from genomic analysis. In the B and R strains, mutations in the *cag4* and gene encoding vacuolating cytotoxin domain-containing protein were detected, which have been shown to contribute to virulence in *H. pylori* and promote bacterial survival [39, 40]. Cag4, which is also known as Cagγ (*hp0523*), refers to one of the proteins encoded by the cag pathogenicity island (cagPAI) [41]. The cagPAI is a genomic region found in *H. pylori* that has been linked to enhanced virulence. The cagPAI gene encodes a type IV secretion system (T4SS), a complex molecular system that allows the bacterium to directly inject bacterial proteins into host cells [42]. CagA (Cytotoxin-associated gene A) protein, Cag4/Cagγ, and other proteins expressed by numerous genes within this region are among the proteins encoded by cagPAI [43]. The putative peptidoglycan hydrolase Cag4/Cag protein is part of the T4SS and is essential for CagA protein secretion and delivery into host gastric epithelial cells [44]. Once inside the host cells, the CagA protein can disrupt a variety of biological processes, altering host cell signalling and causing inflammation which contributes to the development of gastritis, peptic ulcers, and potentially gastric cancer [45]. The vacuolating cytotoxin domain-containing protein, which is also known as FaaA protein from a representative *H. pylori* strain (J99), has been found to improve *H. pylori* colonisation capacity in animal models, and transcription of each gene is elevated in the gastric environment relative to the level of transcription during bacterial growth in vitro [46]. The VacA-like proteins of *H. pylori* are found on the bacterial surface, while the FaaA protein is found on the flagella [47]. A study found that the *faaA* mutant mislocalized the flagella and reduced bacterial mobility [48]. Additionally, SNV mutations was also noted in gene encoding Sel1 repeat family protein in the B and R isolates which is involved in signal transduction pathways between eukaryotes and bacteria [49]. The interactions between bacterial and eukaryotic host cells are thought to be mediated by bacterial Sel1-like repeat (SLR) [50]. Five of the nine secreted proteins from *H. pylori* (HcpAD, HcpA, HcpE, HcpB, and HcpC) folds into a stable three-dimensional structure composed of six disulfides bridged SLRs [51]. These proteins are known to trigger an immune response, causing inflammation [52]. Likewise, Newton et al*.* [53] has noted that Sel1 repeat protein as the virulence determinant of *Legionella pneumophila* which influences vacuolar trafficking. Furthermore, SNV mutations were found in *rsmH* genes among the B and R isolates, which have been linked to antibiotic resistance. Interestingly, mutations in the 16S RNA methyltransferase family (which includes the *rsmH* gene) have been shown to confer aminoglycoside resistance in aerobic Gram-negative bacteria [54, 55]. *Helicobacter heilmannii* isolates with high MIC against neomycin have been shown to have a SNV in the ribosomal RNA small subunit methyltransferase H (*RsmH*) gene [56].

The association between enhanced virulence and resistance development in bacteria is a complex and multifaceted topic. While they are distinct characteristics, there are scenarios where enhanced virulence and resistance development may be interconnected or even co-selected under certain circumstances [57]. Acquiring antibiotic resistance in bacteria may be advantageous for their survival and enhance their virulence [58]. Therefore, *H. pylori* may simultaneously enhance its virulence through exposure to clarithromycin [59]. To combat the emergence and spread of both virulence and resistance in bacteria, it is critical to promote responsible antibiotic use, implement infection prevention measures, monitor resistance patterns, and conduct additional research to understand the underlying mechanisms and interactions between these two traits [60]. Therefore, mutations that occur in R isolates compared to S isolates suggests that mutations are probably involved in antibiotic resistance. However, if mutations that occur in B isolate compared to S isolate, it may not be directly linked to resistance, but it may condition the organism to develop antibiotic resistance.

The development of antibiotic resistance is closely associated with the formation of biofilms in bacterial populations. The biofilm matrix provides protection and shelter to the bacteria within, making them highly resistant to the effects of antibiotics [61]. The continuous presence of increasing concentrations of antibiotics within biofilms can lead to adaptive resistance [62]. The biofilm mass of *H. pylori* may be seen after 3 days of in vitro incubation [63, 64] and can last for up to 7 days under different culture conditions [24, 65–67]. However, some of our samples took longer time to form biofilm and we were unable to see any visible biofilm within 3 days; as a result, we left them for 7 days. The results of this study showed that *H. pylori* produced more biofilm as they developed resistance against clarithromycin. Moreover, bacteria in the biofilm may undergo genetic changes to become more resistant to the specific antibiotics present [68]. It is interesting to note that both B and R isolates have SNV mutations in several genes (*hypE*, *hypF*, and cag pathogenicity island) associated to the development of biofilms. Hydrogenase activity in *H. pylori* is mediated by *hypE* and *hypF*, both of which have been shown to contribute to biofilm formation [63, 69]. A cag pathogenicity island protein is one of the proteins that have been identified as being frequently present with strains that form good biofilms. It has been determined that the CagA protein, which is encoded by the Cag pathogenicity islands, is induced in *H. pylori* biofilms [61]. The Cag pathogenicity island may have a substantial impact on the establishment of the *H. pylori* biofilm. CagA and the cag pathogenicity island may be implicated in the production of *H. pylori* biofilms through their influence on bacteria-bacteria interactions, in addition to their function in bacteria-host interactions [61, 63, 70]. It is important to note that bacterial infections generated by biofilms are frequently more difficult to treat than infections caused by planktonic (free-floating) bacteria [71]. Therefore, researchers are looking at several strategies to combat biofilm-associated antibiotic resistance, including the development of new antimicrobial agents, the use of combination therapy, and the development of biofilm-disrupting techniques. For better treatment outcomes and to solve the problem of worldwide antibiotic resistance, it is essential to comprehend the characteristics of biofilms and how they contribute to antibiotic resistance [72].

## Conclusions

In conclusion, the clarithromycin-sensitive *H. pylori* isolates with the A2143G mutation were successfully induced to be resistant and numerous genes were subjected to genetic reprogramming in response to increasing concentration of clarithromycin. Furthermore, antibiotic exposure may reprogram certain genes such as genes encoding Cag4/Cagγ protein, vacuolating cytotoxin domain-containing protein, sel1 repeat family protein, and *rsmh* gene which could possibly increase the likelihood of antibiotic resistance development and enhances virulence factor in *H. pylori.* Therefore, further studies are required to elucidate these genes mechanisms in antibiotic resistance in *H. pylori* which will help in improving *H. pylori* eradication and develop a new treatment for *H. pylori* infection.

### Supplementary Information

**Additional file 1: Table S1.** Genes with the highest rate of mutation in response to clarithromycin. The corresponding S isolates were used as reference.

**Additional file 2: Table S2.** The MICs and the concentration of clarithromycin used during the induction of clarithromycin resistance for each passage.

**Additional file 3: Figure S1.** Schematic diagram of clarithromycin resistance induction in* H. pylori *sensitive strains.

## Data Availability

All data are available without restriction. Researchers can obtain data by contacting the corresponding author. All data generated or analysed during this study are included in this published article.

## Abbreviations

BHI: Brain Heart Infusion
CagA: Cytotoxin-associated gene A
CA: Chocolate agar
CFU: Colony forming unit
EUCAST: European Committee on Antimicrobial Susceptibility Testing
*H. pylori*: *Helicobacter pylori*
InDels: Insertions or deletions
MIC: Minimum Inhibitory Concentration
OD: Optical density
PBS: Phosphate buffer saline
PPI: Proton pump inhibitor
QC: Quality control
RAPD: Random amplification of polymorphic DNA
SNVs: Single nucleotide variations
T4SS: Type IV secretion system
S: Parental sensitive isolate
B: Breakpoint isogenic isolate
R: Induced resistant isogenic isolate
