# Supplementary figures and images for: Polymorphism of virulence genes and biofilm associated with in vitro induced resistance to clarithromycin in Helicobacter pylori
Source: Gut Pathog. 2023 Oct 28;15:52. doi: 10.1186/s13099-023-00579-4 (PMC10613384; doi:10.1186/s13099-023-00579-4)

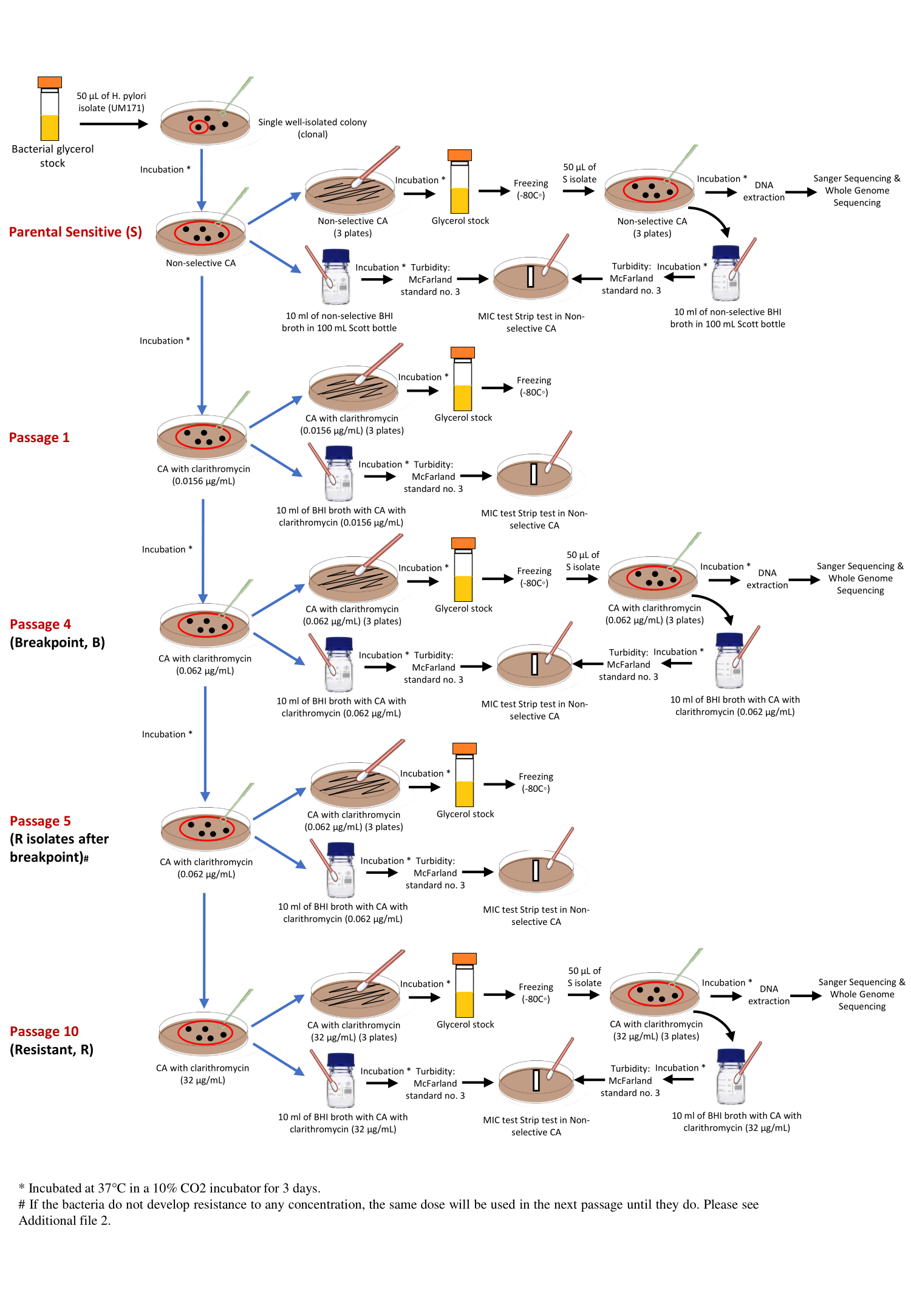

Supplement: Supplementary file 3 — Additional file 3: Figure S1. Schematic diagram of clarithromycin resistance induction in H. pylori sensitive strains. [file 13099_2023_579_MOESM3_ESM.tiff]
